# Hierarchical contribution of individual lifestyle factors and their interactions on adenomatous and serrated polyp risk

**DOI:** 10.1007/s00535-023-02004-8

**Published:** 2023-06-10

**Authors:** Jihee Kim, Kirti Nath, Kurt Schmidlin, Helen Schaufelberger, Christiana Quattropani, Simone Vannini, Sandro Mossi, Miriam Thumshirn, Michael Manz, Lev Litichevskiy, Jiaxin Fan, Oxana Dmitrieva-Posocco, Mingyao Li, Maayan Levy, Primo Schär, Marcel Zwahlen, Christoph A. Thaiss, Kaspar Truninger

**Affiliations:** 1 Microbiology Department and Institute for Immunology, Perelman School of Medicine, University of Pennsylvania, 3610 Hamilton Walk, Philadelphia, PA 19104 USA; 2 Institute of Social and Preventive Medicine, University of Bern, Bern, Switzerland; 3 Clinica Luganese Moncucco, Lugano, Switzerland; 4 Clarunis, University Hospital of Basel, Basel, Switzerland; 5 Department of Biostatistics, Epidemiology and Informatics, Perelman School of Medicine, University of Pennsylvania, Philadelphia, USA; 6 Department of Biomedicine, Genome Plasticity Group, University of Basel, Basel, Switzerland; 7 Gastroenterologie Oberaargau, Langenthal, Switzerland; 8 Clinic of Gastroenterology and Hepatology, Stadtspital Triemli, Zurich, Switzerland

**Keywords:** Adenomatous polyps, Serrated polyps, Individual risk factors, Risk factor interactions

## Abstract

**Background:**

Individual colorectal polyp risk factors are well characterized; however, insights into their pathway-specific interactions are scarce. We aimed to identify the impact of individual risk factors and their joint effects on adenomatous (AP) and serrated polyp (SP) risk.

**Methods:**

We collected information on 363 lifestyle and metabolic parameters from 1597 colonoscopy participants, resulting in over 521,000 data points. We used multivariate statistics and machine-learning approaches to assess associations of single variables and their interactions with AP and SP risk.

**Results:**

Individual factors and their interactions showed common and polyp subtype-specific effects. Abdominal obesity, high body mass index (BMI), metabolic syndrome, and red meat consumption globally increased polyp risk. Age, gender, and western diet associated with AP risk, while smoking was associated with SP risk. CRC family history was associated with advanced adenomas and diabetes with sessile serrated lesions. Regarding lifestyle factor interactions, no lifestyle or dietary adjustments mitigated the adverse smoking effect on SP risk, whereas its negative effect was exacerbated by alcohol in the conventional pathway. The adverse effect of red meat on SP risk was not ameliorated by any factor, but was further exacerbated by western diet along the conventional pathway. No modification of any factor reduced the negative impact of metabolic syndrome on AP risk, whereas increased fatless fish or meat substitutes’ intake mitigated its effect on SP risk.

**Conclusions:**

Individual risk factors and their interactions for polyp formation along the adenomatous and serrated pathways are strongly heterogeneous. Our findings may facilitate tailored lifestyle recommendations and contribute to a better understanding of how risk factor combinations impact colorectal carcinogenesis.

**Supplementary Information:**

The online version contains supplementary material available at 10.1007/s00535-023-02004-8.

## Introduction

Colorectal cancer (CRC) is a heterogeneous disease with phenotypes reflecting underlying molecular pathways [1]. The majority of CRC cases arise from adenomatous polyps (AP), which may progress to advanced adenomas (AA) along the classical pathway, characterized by chromosomal instability and sequential accumulation of genetic mutations [2]. A subtype of CRC cases arises through the serrated pathway with serrated polyps (SP) as precursors. SP encompass hyperplastic polyps (HP), traditional serrated adenomas, and sessile serrated lesions (SSL, formerly designated as sessile serrated adenomas) [3]. Each SP subtype has a distinct malignant potential, with HP considered to harbor no risk of malignancy and SSL representing the main precursors in the serrated pathway. Tumors developing along the serrated pathway frequently display microsatellite instability, the CpG island methylator phenotype (CIMP) and genetic mutations different from the classical pathway [3, 4]. Distinct clinical and molecular phenotypes suggest etiological heterogeneity, with different genetic backgrounds and environmental exposure contributing to carcinogenesis.

Several studies have identified various anthropometric, lifestyle, dietary, and pharmacological factors to modulate risk of CRC and colorectal polyps [3, 5–7]. While variables related to AP risk are well documented, studies analyzing the association between risk factors and SP are limited, given the relatively new concept of this alternative pathway [5]. In addition, only few studies have specifically evaluated risk factors associated with SSL [8]. A meta-analysis reported smoking and alcohol intake as risk factors for SP and SSL, whereas BMI, red meat, and fat intake increased SP, but not SSL risk, while non-steroidal anti-inflammatory drugs (NSAIDs) appeared protective for SP and SSL [5]. Regarding unmodifiable risk factors and in contrast to AP, older age and male gender are not risk factors for SSL [3, 6]. Thus, there is a partial overlap of the AP and SP/SSL risk profiles, which might explain that roughly half of patients with SSL harbor synchronous AP.

Human lifestyles are characterized by the complex interaction of behavioral factors whose collective effects determine the individual risk for disease development. Most previous studies assessed the effect of individual factors or a priori defined lifestyle indices on CRC risk, as proposed by the American Heart Association [9]. By contrast, evidence regarding the combinatorial impact of several individual lifestyle components on colorectal polyp risk is scarce [10, 11]. Here, we aimed to identify the hierarchical structure of various well-known risk factors, as well as their joint effects on early and advanced polyps of both classical and alternative pathways. We present the clinical data from the Swiss Epigenetic Colorectal Cancer Cohort (SWEPIC) study, initiated to investigate (epi)genetic instability and their underlying causes in colorectal carcinogenesis. We used computational and statistical methods to integrate data sets from deeply characterized study participants in order to develop a model of risk factor interactions and risk factor mitigation by lifestyle changes.

## Methods

### Study population

SWEPIC is a prospective colonoscopy-based study conducted in Switzerland. Patient recruitment was initiated in October 2014 and ended in January 2017. Study participants aged 40 years or older were referred for colonoscopy from their family doctors for various reasons (1163 screening colonoscopy, 126 surveillance colonoscopy, 201 diagnostic colonoscopy, 107 unknown). Study participants underwent their first colonoscopy, except for the indication of surveillance. All patients were included and analyzed in this study, including the subjects undergoing surveillance colonoscopy. Ineligibility for recruitment in SWEPIC study was defined as having previous colorectal surgery, familial adenomatous polyposis, hereditary non-polyposis colorectal cancer and inflammatory bowel disease (IBD) or inflammation suggestive of IBD during colonoscopy. Colonoscopy was conducted during routine schedule by experienced gastroenterologists (H.S., C.Q., S.V., S.M., M.T., M.M., K.T.; each > 10,000 colonoscopies) at the Gastrocentro Lugano, Claraspital Basel, and Gastroenterologie Oberaargau. All polyps detected in this study were endoscopically removed and histologically examined. Informed consent was obtained from all participants and the study was approved by the Ethikkommission Nordwest- und Zentralschweiz, Basel, Switzerland (EK 276/13).

### Data collection

Detailed information on colonoscopy and histological findings of removed polyps were reported. Information on demographics, BMI, smoking, medication use, physical activity, nutrition, medical history, family history of CRC and other lifestyle factors were obtained by interview with a study nurse (696 subjects) or self-administered questionnaire (901 subjects), depending on local availability.

Polyps were histologically classified according to 5th edition of the WHO classification of tumors of the digestive system [12]. For subsite classification, polyps were defined as proximal when located proximal to the splenic flexure, distal when located in the descending or sigmoid colon, and those in the rectum as rectal. AP were defined as non-advanced if 1–2 tubular adenomas < 10 mm were found, and as advanced adenomas if ≥ 10 mm, or if villous component or high-grade dysplasia were present. Educational attainment was grouped into three categories: “compulsory education” (compulsory schooling up to 9 years of education or less), “secondary education” (high school, teachers training colleges, vocational education), and “tertiary education” (all university undergraduate, post-graduate, higher professional training). Family history of CRC was classified as positive if at least one CRC had been diagnosed in a first-degree relative. BMI was calculated from height and weight measured immediately before colonoscopy.

Smoking was classified as never, former (one cigarette daily for at least 1 year, quit more than a year prior to study enrollment) and current (one cigarette daily for at least 1 year, including the year prior to study enrollment). A pack year was defined as having smoked 20 cigarettes every day for 1 year. Use of baby aspirin (100 mg) or NSAID was categorized according to length of intake (> 5 years), but not on frequency of consumption. According to the NCEP-ATP-III definition, metabolic syndrome was diagnosed if three or more of the following five criteria were met: waist circumference (men > 102 cm, women > 88 cm), blood pressure over 130/85 mm Hg (or antihypertensive treatment), fasting triglyceride level over 1.7 mmol/l (or treatment of high triglyceride level), fasting high-density lipoprotein of less than 1.05 mmol/l in men and less than 1.25 mmol/l in women, and fasting glucose level of 5.6 mmol/l or higher (or diabetes mellitus diagnosis) [13, 14]. Absence of visceral adiposity was defined as waist circumference < 102 cm in men and < 88 cm in women.

Physical activity was self-reported using the long form of the international physical activity questionnaire (IPAq) and was defined as low, moderate or vigorous (“Guidelines for the data processing and analysis of the International Physical Activity Questionnaire” https://sites.google.com/site/theipaq/scoring-protocol).

Information on nutrition was collected with a short form of a previously validated questionnaire to assess both frequency, portion size and number of portions of 52 food items during last 4 weeks [15].

### Statistical analysis: dietary factor assessment

Patients were scored on their overall consumption of various common food items and macronutrient consumption was calculated from these food items based on consensus nutritional information for each food item. Next, food items were grouped into either prudent or western and either anti-inflammatory or pro-inflammatory based on previous literature describing canonical dietary patterns [16]. Food items were further grouped according to NOVA classification depending on the degree of industrial processing [17]. The proportion of diet considered either western or pro-inflammatory was used to create western and inflammatory indices, which were sometimes considered as a numerical quantity and otherwise grouped with threshold values to create factors. Out of 1597 randomly recruited patients, dietary information was collected from 1493. All analyses involving dietary factors were performed on this subset of patients.

### Statistical analysis: mixed factor analysis

We used mixed factor analysis as a dimensionality reduction strategy in order to identify data dimensions with the strongest association with clinical observations. Specifically, metabolic syndrome, hypertension, BMI, elevated triglycerides, hyperglycemia, abdominal obesity, diabetes, age, smoking quantity, smoking status, gender, inflammatory index and western index were considered for mixed factor analysis, which was utilized to apply a principal component analysis on both quantitative and qualitative variables in the dataset. Analysis was conducted using R packages ‘FactoMineR’ (2.4) and ‘factoextra’ (1.0.7) [18–20]. Non-redundant dimensions were selected as meta-factors and used in subsequent analysis.

### Statistical analysis: odds ratio analysis

Odds ratios calculated for various clinical diagnoses were conducted using the R package ‘epiR’ (2.0.19) and ‘oddsratio’ (2.0.1). Odds ratios were calculated using Wald 95% confidence intervals. Significance for each odds ratio was calculated from logistic regression models, adjusted for age, gender and BMI.

### Statistical analysis: random forest

Random forest analysis was conducted using the R package ‘randomForest’ (4.6–16). Parameters were tuned using ‘tidymodels’ (0.1.2).

### Statistical analysis: logistic regression

Binomial logistic regression was used to evaluate each risk factor while controlling for age, gender and BMI.

### Statistical analysis: likelihood ratio tests for hierarchical risk factor analysis

Likelihood ratio tests were conducted using the R package ‘lmtest’ (0.9–40), adjusting for age and gender. Two risk factors were considered to have a significant combined effect when *p* < 0.1.

## Results

### Demographic and clinical characteristics of the study population

**Fig. 1:**
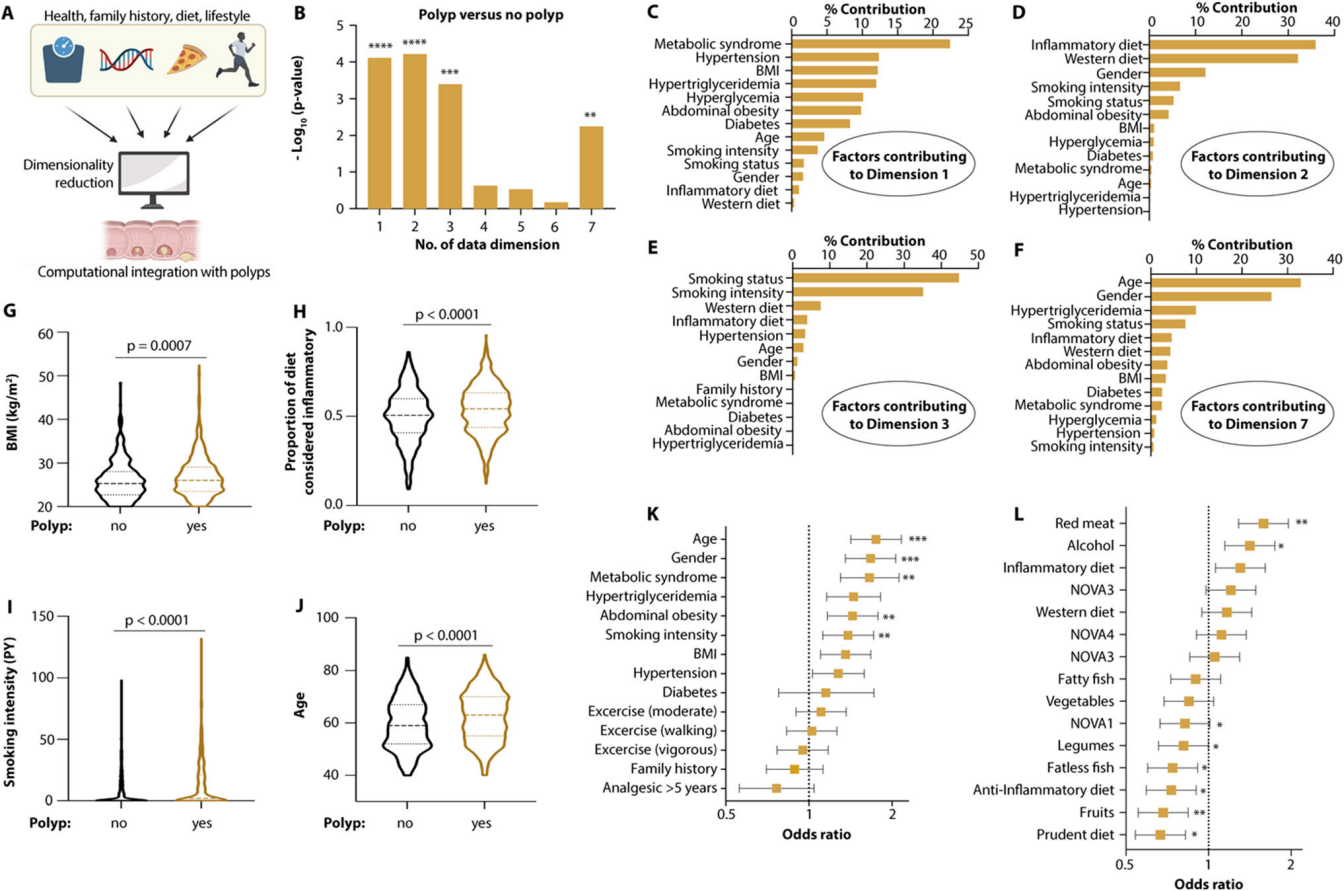
Risk factor characterization of any polyp. **A** Schematic of computational analysis of polyp-associated risk factors. **B** Statistical associations of individual mixed factor analysis dimensions with polyp detection. **C**–**F** Relative contribution of each risk factor to mixed factor analysis dimensions that distinguish individuals with and without polyps. **G**–**J** Comparison of various risk factors associated with polyp detection. **K**, **L** Odds ratio plots with Wald 95% confidence intervals for various dietary and lifestyle risk factors. Asterisks indicate results of the significance of logistic regression models of each risk factor, adjusted for age, gender and BMI. Ordinary one-way ANOVA with Turkey’s multiple comparisons (**B**), two-tailed unpaired *t* tests (**G**–**J**), Benjamini–Hochberg-adjusted p-values from logistic regression models (**K**, **L**). *p < 0.1, **p < 0.05, ***p < 0.001, ****p < 0.0001 was considered significant

The cohort comprised 1597 subjects who fulfilled the inclusion criteria and provided informed consent to participate in the SWEPIC study (Supplementary Results, Fig. 1A). Table 1 shows the basic characteristics of study participants. The median age was 61 years (range 40–86; Supplementary Results, Fig. 1B) and 49% of participants were women. We documented polyps in 896 (56%) study participants (63% in men, 51% in women). AP, AA, HP, and SSL were detected in 596 (37%), 124 (8%), 409 (26%), 174 (11%) subjects respectively (Supplementary Results, Fig. 1C). The proportion of patients with SSL harboring also AP was 79/174 (45%). Study participants were more likely to harbor AP (26%), AA (4%) and SSL (10%) in the proximal colon compared to the distal colon (AP: 18%; AA: 3%; SSL: 2%) or rectum (AP: 5%; AA: 1%; SSL: 0.3%), while more subjects had SP in the rectum (12%) and sigmoid colon (11%) compared to the proximal colon (6%) (Supplementary Results, Fig. 1D).

**Table 1:** Baseline Characteristics of participants in the SWEPIC cohort

Characteristics	No Polyp group	Polyp group		
		AP only	SP only	AP and SP
Number of participants	701	378	300	218
Age in years, mean (SD)	59.5 (9.72)	64.0 (9.70)	59.0 (8.54)	64.5 (8.72)
Gender, *n* (%)				
Male	313 (44.7)	229 (60.5)	144 (48.0)	136 (62.4)
Female	388 (55.3)	149 (39.5)	156 (52.0)	82 (37.6)
Advanced polyps, *n* (%)				
Advanced AP		78 (20.6)		
Sessile serrated lesion			95 (31.7)	
Both				22 (10.1)
Family history of colorectal cancer				
Yes (%)	181 (25.8)	86 (23.2)	78 (24.9)	57 (25.3)
Smoking status, *n* (%)				
Never	345 (49.2)	159 (42.1)	128 (42.7)	89 (40.8)
Former	221 (31.5)	139 (36.8)	97 (32.3)	78 (35.8)
Current	80 (11.4)	37 (9.79)	64 (16.9)	44 (20.2)
Unknown	55 (7.85)	42 (11.1)	11 (2.91)	7 (3.21)
Smoking intensity, mean (SD)				
Pack years	8.10 (14.4)	11.6 (18.7)	11.6 (16.2)	15.4 (20.6)
Metabolic syndrome, *n* (%)				
Yes	151 (21.5)	109 (28.9)	69 (23.0)	91 (41.6)
Body mass index (kg/m^2^), mean (SD)				
Mean (SD)	25.7 (4.48)	26.5 (4.67)	26.1 (4.62)	27.7 (4.96)
Physical activity (min/week), mean (SD)	824 (566)	822 (573)	810 (521)	809 (551)
Aspirin or NSAID > 5 years, *n* (%)				
Yes	142 (20.3)	69 (18.2)	59 (19.7)	44 (20.2)
Nutrition, *n* (%), (SD)				
Prudent	25.9 (0.110)	23.2 (0.094)	24.1 (0.101)	22.5 (0.094)
Western	24.3 (0.110)	25.0 (0.099)	24.3 (0.099)	25.3 (0.097)
Anti-inflammatory	25.4 (0.103)	23.0 (0.084)	23.6 (0.094)	22.3 (0.087)
Pro-inflammatory	39.3 (0.134)	41.5 (0.122)	40.0 (0.124)	43.2 (0.121)

### Lifestyle factors

Lifestyle characteristics of no polyp and various polyp groups are shown in Table 1. The BMI was normally distributed across the cohort with a mean of 26.23 kg/m^2^ and a standard deviation of 4.66 kg/m^2^ (Supplementary Results, Fig. 1E). Of patients who reported their smoking status, 714 identified as having never smoked, 531 responded that they were former smokers, and 225 were current smokers (Supplementary Results, Fig. 1F). A large proportion of patients fulfilled at least one of the criteria defining metabolic syndrome (Supplementary Results, Fig. 1G). Mean physical activity was 116.97 min/day with a standard deviation of 79.53 min/day, which included moderate activities such as walking (Supplementary Results, Fig. 1H).

### Dietary factors and dietary patterns

The majority of participants had a diet rich in carbohydrates and approximately equal consumption of fat and protein, with grain and dairy being the major dietary components (Supplementary Results, Fig. 1I–L). Dietary patterns were classified as inflammatory, anti-inflammatory, western, or prudent [16], which showed a normal distribution in the study cohort (Supplementary Results, Fig. 1J).

### Risk factors common to any polyp

**Fig. 2:**
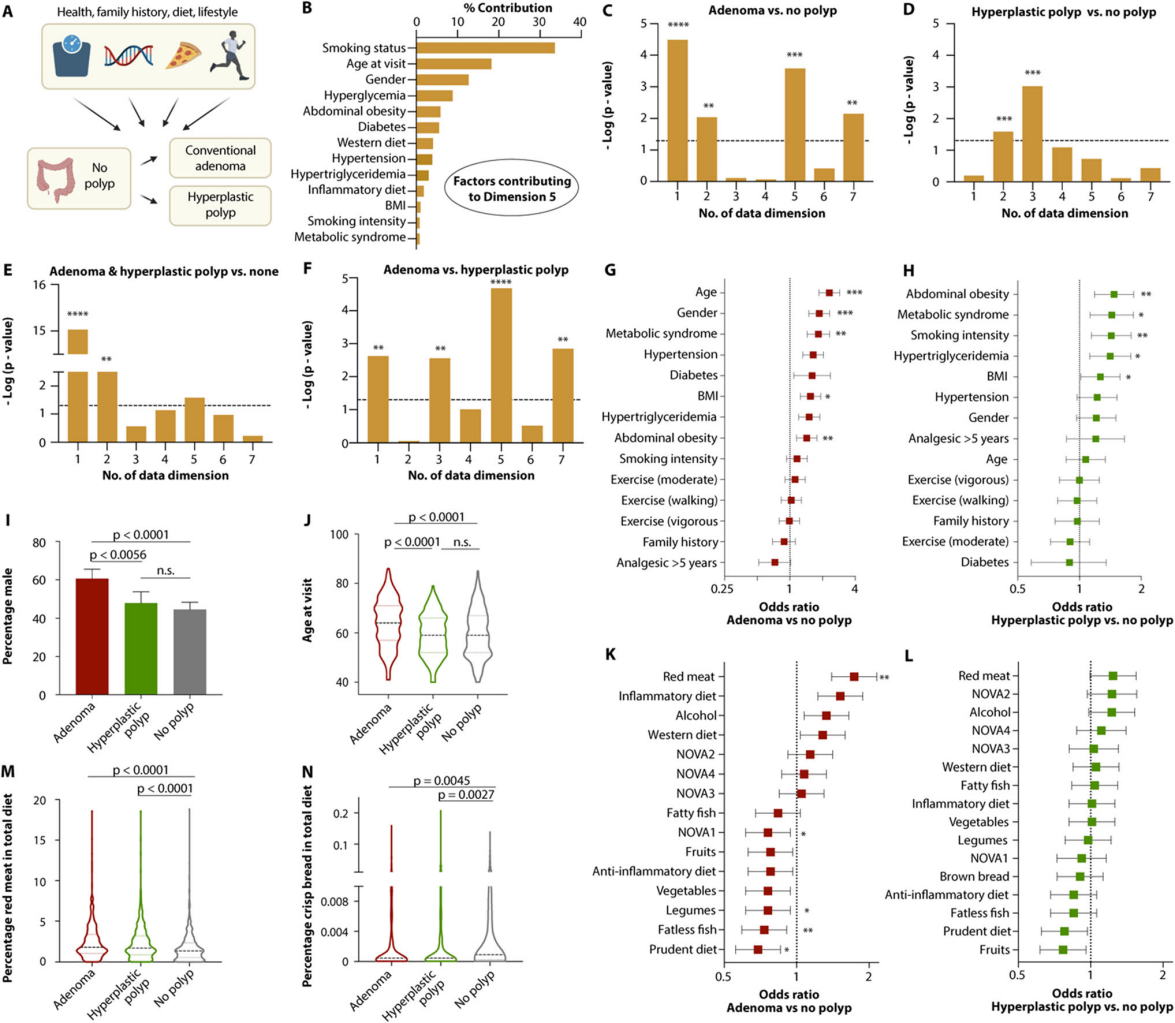
Pathway-specific risk factor characterization. **A** Schematic of computational analysis of risk factors influencing the development of adenomatous versus serrated precursor lesions. **B** Percent contribution of each risk factor to dimension 5 of the mixed factor model. **C**–**F** Results of unpaired t-test comparing the dimension coordinate of each dimension for individuals with conventional adenomas (**C**), hyperplastic polyps (**D**), both types of lesions (**E**), or between the two types of precursor lesions (**F**). **G**, **K** Odds ratio plots with Wald 95% confidence intervals for risk factors for the conventional (**G**) and alternative (**H**) precursor pathway. Asterisks indicate results of the significance of logistic regression models of each risk factor, adjusted for age, gender and BMI. **I**, **J** Comparison of male sex (**I**) and age (**J**) in cohorts with colonoscopy-based detection of adenomas, hyperplastic polyps, or neither. **K**, **L** Odds ratio plots with Wald 95% confidence intervals for conventional (**K**) and alternative (**L**) precursor pathway risk factors. Asterisks indicate results of the significance of logistic regression models of each risk factor, adjusted for age, gender and BMI. **M**, **N** Comparison of red meat consumption (**M**) and crisp bread consumption (**N**) in cohorts with colonoscopy-based detection of adenomas, hyperplastic polyps, or neither. Ordinary one-way ANOVA with Turkey’s multiple comparisons (**C**–**F**, **I**, **J**, **M**, **N**), Benjamini–Hochberg-adjusted p-values from logistic regression models (**G**, **H**, **K**, **L**). *p < 0.1, **p < 0.05, ***p < 0.001, ****p < 0.0001 was considered significant. Error bars (*I*) indicate mean with 95% confidence intervals

To identify variables among the more than 521,000 features collected across the entire cohort that associated with colonoscopy findings, we used a dimensionality reduction strategy, coupled to statistical assessment of risk factor impact (Fig. 1A). Mixed factor analysis produced seven non-redundant dimensions (Fig. 1B; Supplementary Results, Fig. 2A). Dimensions were considered redundant if they consisted of largely the same factor contributors as another dimension. Four of the seven dimensions were significantly different in individuals harboring any polyp compared to those with no polyps (Fig. 1B). Dimension 1 was largely composed of metabolic syndrome and its defining features, dimension 2 by western and inflammatory dietary patterns, dimension 3 by smoking status and quantity, and dimension 7 was characterized by demographic factors, namely age and sex (Fig. 1C–F). Thus, at the level of global risk factor assessment, our study is consistent with previous findings by identifying a high BMI and abdominal obesity, dietary patterns considered inflammatory, smoking, as well as increased age and male sex as risk factors (Fig. 1G–K; Supplementary Results, Fig. 2B–D). Increasing consumption of dietary components classified as prudent (fatless fish, fruits) lowered the risk for polyps, while the consumption of red meat and alcohol was associated with higher occurrence of polyps (Fig. 1L; Supplementary Results, Fig. 2E–H). In contrast, family history was not a major determinant of overall polyp risk (Fig. 1K).

### Risk factors unique to adenomatous or serrated polyps

We also applied mixed factor analysis to identify polyp subtype-specific variables and factors distinguishing between the classical and serrated pathways (Fig. 2A). Individuals with only AP differed from patients without polyps across dimensions 1 (metabolic syndrome), 2 (diet), 5 (largely defined by a combination of smoking status, age, and gender; Fig. 2B), and 7 (demographics) (Fig. 2C). In contrast, individuals with only HP differed from individuals without polyps across dimension 2 and 3 (smoking) (Fig. 2D). Individuals with synchronous AP and HP differed from the no polyp population across dimensions 1 and 2 (Fig. 2E). Finally, comparing individuals with only AP versus individuals with only SP revealed a significant difference across dimensions 1, 3, 5, and 7 (Fig. 2F). These findings suggest that the risk factor structure is strongly different for both pathways in regard to the canonical risk factors associated with CRC, such as age, sex, and metabolic syndrome (Fig. 2G–I).

**Fig. 3:**
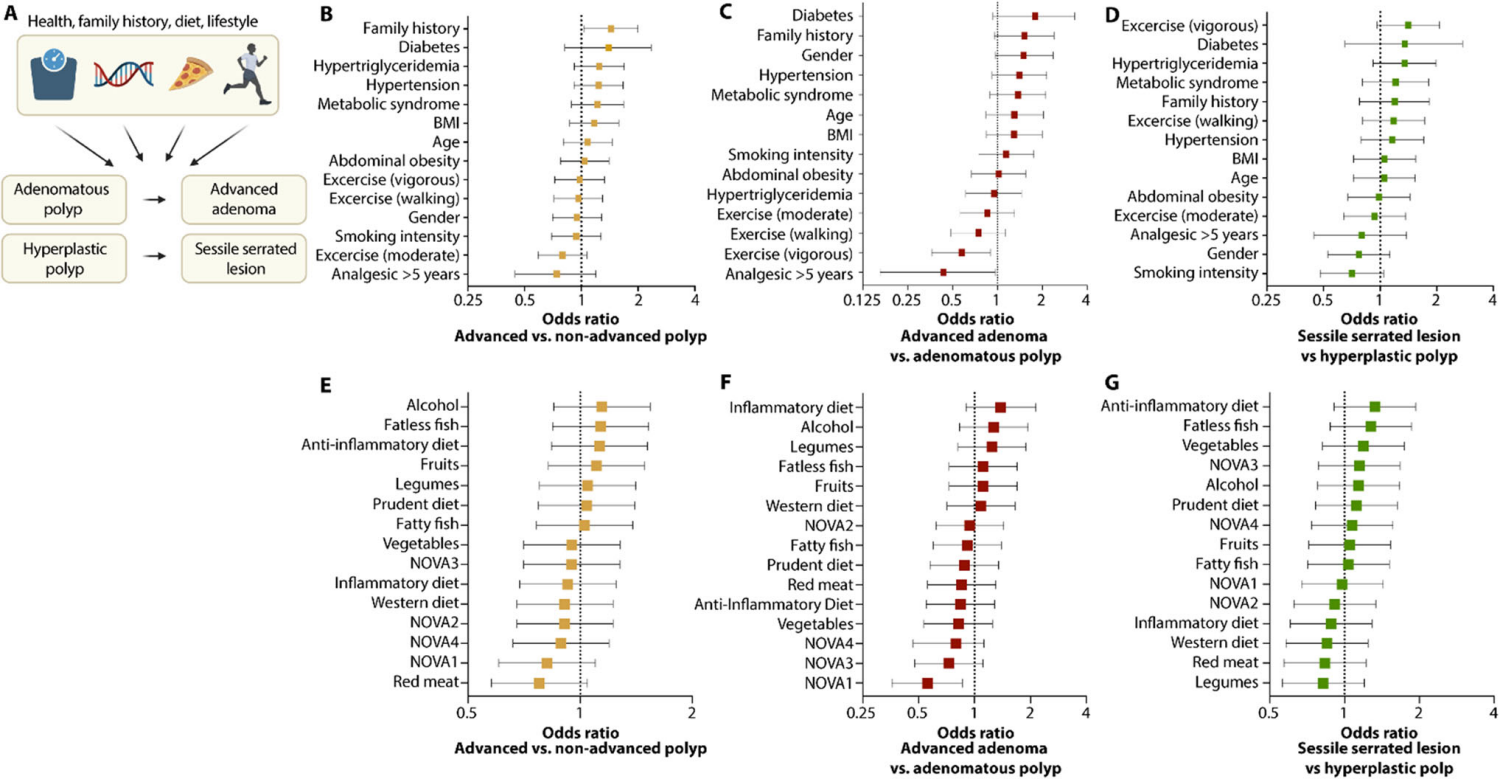
Factors modulating lesion progression along the classical and serrated pathway. **A** Schematic of computational analysis of risk factors influencing the progression of conventional versus alterative precursor lesions to advanced lesion types. **B**–**G** Odds ratio plots with Wald 95% confidence intervals comparing early and late-stage presentation of both pathways combined (**B**, **E**), advanced vs adenomas (**C**, **F**), and hyperplastic versus sessile serrated lesions (**D**, **G**)

We analyzed these patterns further by comparing specific risk factors across the different clinical outcome groups. Interestingly, increased age at visit and male gender only increased the risk of AP, while the presence of metabolic syndrome, abdominal obesity, and elevated BMI were common risk factors in both classical and serrated pathways (Fig. 2G–J). In contrast, smoking intensity (measured in packs per year) was only significantly associated with an increased SP risk (Fig. 2G, H). Of note, neither clinical outcome was associated with family history (Fig. 2G, H). Dietary risk factors were largely similar for polyps of both pathways (Fig. 2K–N; Supplementary Results, Fig. 3A–C), with the consumption of fatless fish possibly being more protective against the development of AP than SP (Fig. 2K, L; Supplementary Results, Fig. 3D).

### Risk factors of progression to advanced polyps

We also assessed the impact of risk factors associated with the progression from early to advanced polyps (Fig. 3A). While limited by sample size, our analysis was able to rank risk factors by their relative contribution to the overall progression to advanced polyp types, the progression from AP to AA, and the progression from HP to SSL. Notably, family history emerged as the most important variable comparing early versus advanced polyps in a pathway-agnostic fashion as well as for the progression from AP to AA (Fig. 3B, C). Contrastingly, in the serrated pathway, progression from HP to SSL was not associated with family history but was affected by diabetes (Fig. 3B, D). Furthermore, vigorous exercise appeared to reduce AA risk (Fig. 3C).

**Fig. 4:**
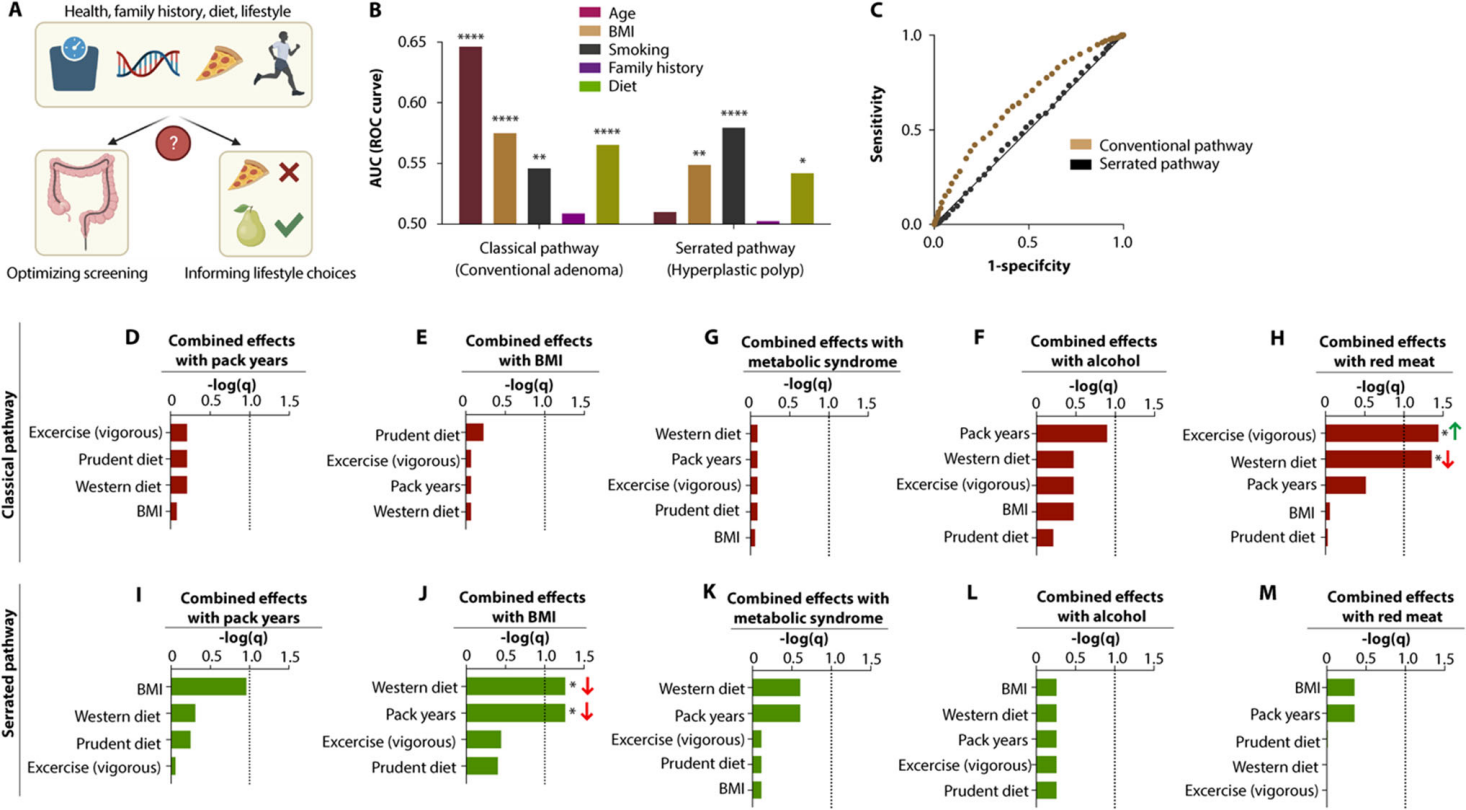
Combinatorial effects of risk factors on detection efficiency and polyp risk modulation. **A** Schematic of strategic approach to optimize screening strategies and lifestyle recommendations based on risk factor contributions. **B** ROC analysis of age, BMI, smoking, family history, and diet for colonoscopy findings in the classical and alternative pathways of precursor lesions. **C** ROC curve for age in the conventional and alternative pathway. **D**–**M** Results of likelihood ratio tests, showing significance of the combined effects of any two risk factors in the conventional (**D**–**H**) and serrated (**I**–**M**) pathways. Arrows indicate recommendations for risk mitigation (red arrows: risk exacerbation, green arrows: risk mitigation). Benjamini–Hochberg-adjusted *p* values from likelihood ratio tests (**D**–**M**); dashed lines represent significance threshold. *p < 0.1, **p < 0.05, ***p < 0.001, ****p < 0.0001 was considered significant

The relative contributions of dietary risk factors likewise differed between the two distinct precursor pathways, with dietary patterns characterized as inflammatory being more associated with the progression from AP to AA (Fig. 3E–G), while the consumption of nuts was more strongly associated with the progression from HP to SSL (Supplementary Results, Fig. 4A–C).

### Machine learning-based risk factor analysis

**Fig. 5:**
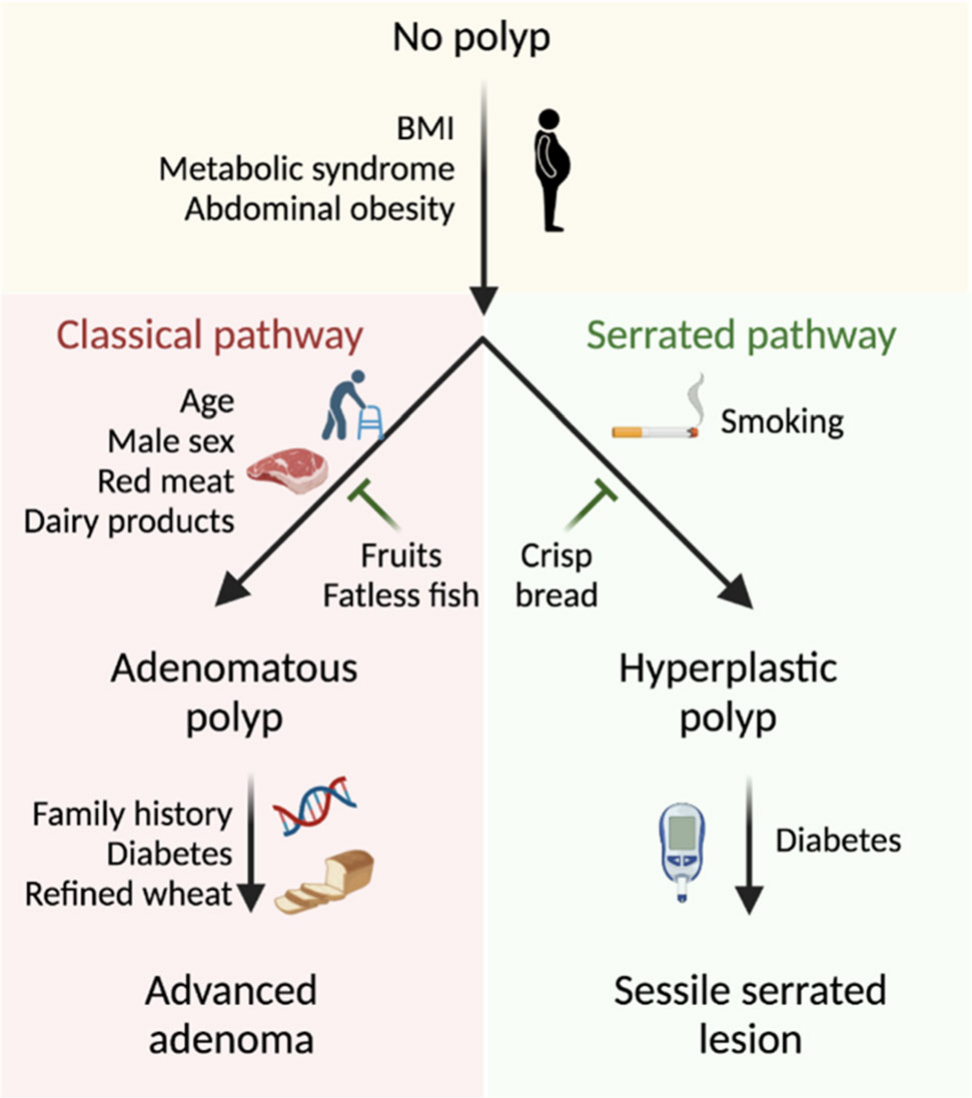
Hierarchical risk factor model for the initiation and progression of adenomatous and serrated polyps

Our analysis suggested subtype- and stage-specific contributions of anthropometric and lifestyle factors on the development of colonic polyps. To verify these results using orthogonal methods, we developed random forest models for each of the major clinical comparisons in using decrease accuracy to proxy each factor’s importance to the respective models. We first validated the model by determining risk factors for the classical or serrated pathways. Indeed, the most important risk factors for the prediction of the presence of any polyp were age at visit, gender, smoking quantity, inflammatory index, and metabolic syndrome (Supplementary Results, Fig. 5A). Similarly, the most important risk factors for the presence of predicting AP were age at visit, gender, body mass index, and metabolic syndrome (Supplementary Results, Fig. 5B). In contrast, the most important factors for predicting the presence of a SP were inflammatory index, smoking history, as well as chronic consumption of aspirin or analgesic, and western index (Supplementary Results, Fig. 5C). Notably, when we trained a model to predict whether a patient has an AP or AA, the most important feature was family history (Supplementary Results, Fig. 5D). Thus, the conclusions obtained by a trained classifier are largely consistent with our results from mixed factor analysis, implying differential potency of individual factors for risk prediction and stratification.

### The impact of lifestyle adjustment on risk modulation

Finally, we sought to probe the utility of these findings for improving lifestyle recommendations to mitigate individual risk (Fig. 4A). Age, which is the most common major criterion for screening colonoscopies, showed strongly differential detection sensitivity and specificity for the classical versus alternative pathway, with a near-random performance for serrated polyps (Fig. 4B, C). In contrast, BMI, smoking status, and dietary patterns were effective as criteria for examination of both the classical and serrated pathways, while information about family history did not perform better than a random model for either pathway (Fig. 4B).

Our approach also enabled us to examine combinations of risk factors and the change in relative risk achieved by altering individual risk factors. We thus examined pairwise combinations of risk factors, their joint impact on AP and SP development, and risk modifications caused by addition or removal of individual risk factors (Fig. 4D–M, Supplementary Results, Fig. 6A–F). Notably, in the classical pathway no change in any risk factor was able to reduce the impact of metabolic syndrome on AP risk (Fig. 4G). In contrast, elevated AP risk due to red meat consumption can be mitigated by reducing the proportion of western diet and vigorous exercise and increasing fatty fish consumption (Fig. 4H, Supplementary Results, Fig. 6D). Significant interactions were also noticed between smoking and alcohol consumption (risk exacerbation), BMI and fruit consumption (risk exacerbation), alcohol and dressing consumption (risk exacerbation), as well as red meat and soft drink consumption (risk exacerbation) (Supplementary Results, Fig. 6A-D).

In the serrated pathway, no change in any risk factor was able the reduce the impact of smoking on HP risk (Fig. 4I). The risk conferred by an elevated BMI was exacerbated in combination with western diet or smoking (Fig. 4J). The consumption of fatless fish and meat substitutes reduced the risk conferred by metabolic syndrome (Supplementary Results, Fig. 6E). In contrast to the classical pathway, no alterations could be made to reduce the dominating effects of red meat consumption (Fig. 4M).

These results collectively suggest that risk factor combinations exert disparate effects on the classical and serrated pathways of colonic precursor lesions.

## Discussion

Recent estimates suggest that a significant fraction of CRC incidence and mortality is preventable by means of lifestyle changes [10], making a deep understanding of environmental risk factors essential for disease prevention and early detection strategies. Herein, we present the clinical data from the SWEPIC study, comprising 1597 unselected individuals undergoing colonoscopy whose lifestyle and environmental exposure were deeply characterized. Using a combination of mixed factor modeling and random forest classification, our data strengthen previous data on the contribution of individual risk factors to different polyp subtypes. Moreover, we dissected interactions of modifiable risk factors whose collective effects better reflect an individual’s exposure and polyp risk (Fig. 5). Our findings may improve CRC prevention and provide potential new insights into the mechanisms through which risk factors affect colorectal carcinogenesis.

The new findings derived from this study may have several important clinical implications and provide the basis for the systematic exploration of pathway-related risk factor interactions aimed at optimizing CRC prevention. While most previous studies have looked at the effects of individual risk factors in isolation, our study highlights several unexpected features of pathway-specific interactions of modifiable risk factors. While certain lifestyle factors exert a dominant effect, the impact of other variables was modifiable. For example, smoking cessation without reducing BMI may mitigate risk for SP, but not AP, whereas more physical activity and reduced western diet consumption can only reduce risk for AP, but not SP. Exercise and western diet may modulate the effect of red meat intake on AP, but not SP risk. In contrast, no change in any risk factor was able to mitigate the negative impact of metabolic syndrome on AP risk. Hence, our data suggest that the contribution of individual risk factors is not always absolute but might be modulated by the presence of additional variables in the same individual along the classical as well as the serrated pathway. It is reasonable to assume that tailored recommendations for lifestyle modifications not only are more efficient in CRC prevention, but likely better convince an individual about the importance of an intervention. Beside better understanding of risk factors, stratifying the population by CRC risk offers the potential to improve the efficiency of screening. Risk stratification for screening is currently based primarily on age, which was only associated with polyp detection in the classical pathway in our study. Furthermore, information about CRC family history may not provide added benefit for optimized screening. Rather, our findings indicate that the success of CRC screening may be improved by incorporating other variables, such as BMI, smoking status, and dietary patterns. Risk adapted secondary prevention by individualized screening approaches is particularly important, since multiple professional societies have lowered the recommended age for CRC screening to 45.

The results from this study provide also new pathogenetic insights. For instance, smoking alone is a critical risk factor for the serrated pathway, while for the development of adenomatous polyps, smoking only associates with disease risk in combination with alcohol consumption. This finding further emphasizes the notion that lesion development along the classical pathway might be driven by environmental modulators of age-associated defects, whereas polyp formation along the serrated pathway is independent of the age-associated decline in tissue function, but equally susceptible to environmental carcinogens at any age.

Second, given the observational nature of our data, putative mechanistic directions should be interpreted with caution. Nevertheless, the differential involvement of risk factors in the initiation and progression of colorectal polyps may contribute to our understanding of their molecular etiology and pathogenesis. Our finding that CRC family history was not associated with the presence of AP, but strongly linked with AA indicates that environmental exposure dominates genetic susceptibility at early stages, while genetic factors may determine the rate of progression to advanced lesions. In contrast, our analysis indicates that the classical pathway is a bona fide age-associated process, in which the accrual of environmental exposures contributes to progressive accumulation of genetic and epigenetic alterations. The generation of AP can, therefore, be considered a result of declining tissue maintenance functions, such as DNA repair, with advanced age that is modulated by exposure interactions.

In contrast, and in line with previous reports, age was not associated with an increased risk for alternative pathway polyps in our study, indicating that the molecular processes associated with the genesis of SP are age-independent and equally responsive to environmental exposure across the lifespan. BRAF-V600E is the key somatic mutation in generation of the serrated pathway, and the majority of HP and SSL harbor this mutation [21, 22]. Hence, colonic epithelial cells are equally susceptible to acquisition of the BRAF-V600E across the lifespan. Whether smoking, the most critical risk factor associated with HP risk, directly or indirectly induces BRAF-V600E (e.g., advantage for colonic epithelial cells harboring mutant BRAF) is unknown.

The strengths of this study include the prospective collection of data on polyps, histology, demographics, lifestyle and nutrition. We were able to obtain all data during routine clinical gastroenterological practice rather than in a specific study population. The high detection rate of polyps (57%), AP (37%), AA (8%) and SP (32%) reflects the quality of the endoscopists generating the SWEPIC cohort. Diagnostic histological accuracy of all removed polyps was provided by expert pathologists. In addition, this is the first study to evaluate the impact of dietary patterns on AP and SP risk, in conjunction with assessing the vast majority of modifiable risk factors, thereby achieving a comprehensive picture of environmental contributions to both CRC pathways. Moreover, while previous studies simply calculate a healthy lifestyle index by summing multiple binary lifestyle factors, this study analyses the interaction of different variables, thus enabling risk-adapted guidelines for colonoscopy screening criteria. Finally, our approach with multivariate statistics and machine-based tools revealed similar results, which strengthen our findings.

Among the limitations to our study is the fact that study population was too small to achieve the statistical power needed to analyze subsite-specific associations for the various risk factors and to determine location-specific exposure susceptibility. We also cannot exclude selection bias, i.e., participants of our cohort may have different characteristics and behavior than subjects who did not receive colonoscopy. In addition, our cohort included non-screening individuals. Our analysis was based on a cross-sectional study design and assessment of lifestyle, personal and family history of various diseases, and medication use was based on questionnaire and self-reported information and is subject to information error. Furthermore, we incorporated polyp numbers only categorically (≤ 2, 3–4, 5–9, ≥ 10) and, therefore, could not analyze the precise polyp burden per subject and subsite.

In conclusion, our analysis highlights the importance of polyp subtype-specific comprehensive risk factor assessment to investigate pathway-related risk factor combinations, which better reflect real-life behavior. Our study indicates that the differential contribution of genetic and environmental factors to the pathogenesis of colorectal polyps along the classical and serrated pathways enables the optimization of screening criteria and the personalization of lifestyle recommendations.

## Supplementary Information

Below is the link to the electronic supplementary material.

Supplementary file1 (DOCX 1351 kb)

## Data Availability

The datasets used and analyzed during this study are available from the corresponding author on reasonable request.

## Abbreviations

AP: Adenomatous polyps
SP: Serrated polyps
HP: Hyperplastic polyps
SSL: Sessile serrated lesions
AA: Advanced adenomas
CRC: Colorectal cancer
BMI: Body mass index

## Declarations

### Gratitude

We would like to express our gratitude to all study participants and the staff of the involved clinics for their valuable support. We also thank Christine Brombach for serving as scientific advisor for assessment of nutrition.
