# Supplementary material for: Hierarchical contribution of individual lifestyle factors and their interactions on adenomatous and serrated polyp risk
Source: J Gastroenterol. 2023 Jun 10;58(9):856–67. doi: 10.1007/s00535-023-02004-8 (PMC10423128; doi:10.1007/s00535-023-02004-8)
Supplement: Supplementary file 1 — Supplementary file1 (DOCX 1351 kb) [file 535_2023_2004_MOESM1_ESM.docx]

**Supplementary Material**

**
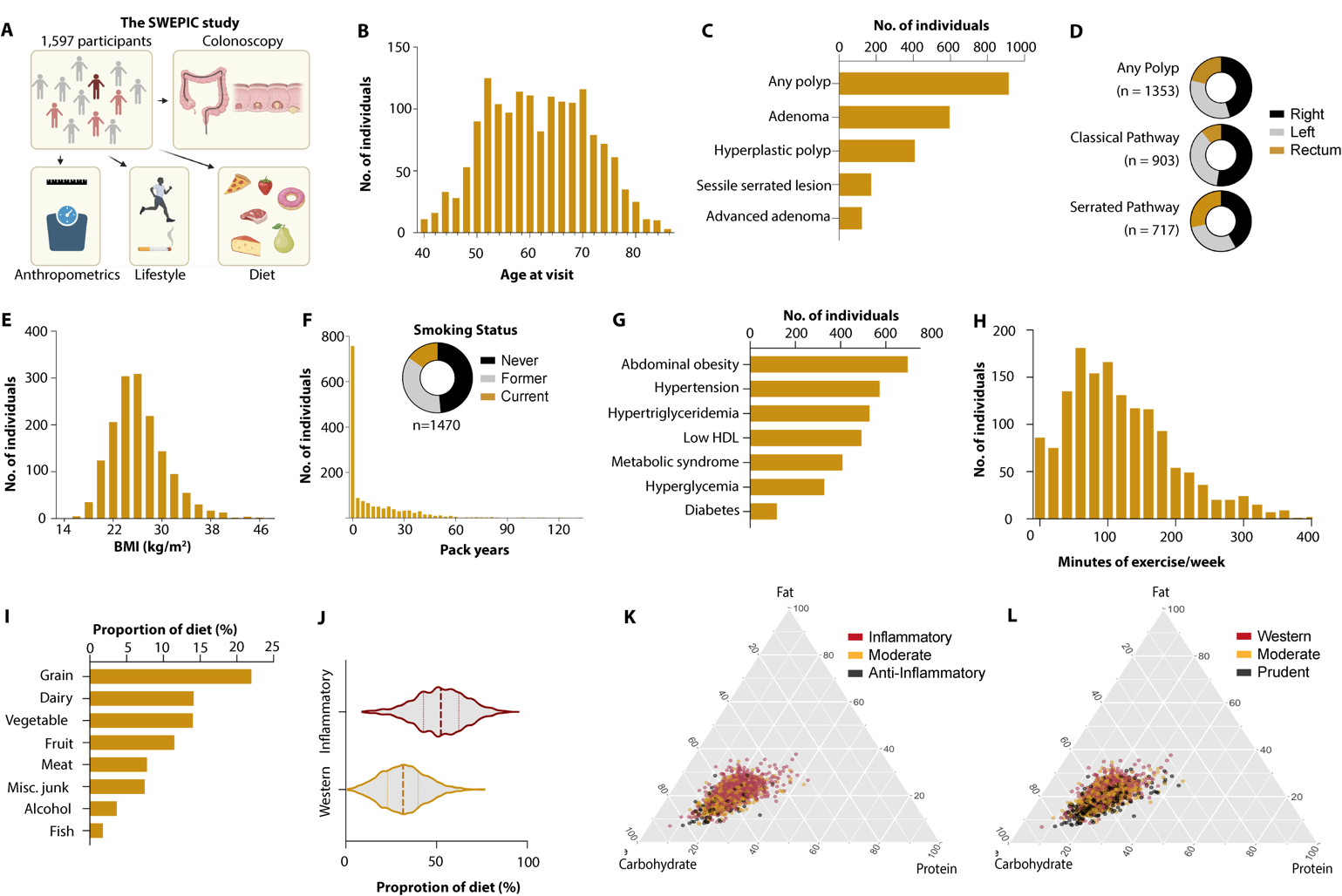
**

**Supplementary Results Figure 1. Baseline characteristics of participants and polyps in the SWEPIC cohort.**

(A) The clinical cohort contained 1,597 participants whose clinical, behavioral, and dietary characterization were analyzed across various dimensions.

(B) Age distribution.

(C, D) Polyp type (C) and location (D).

(E-H) BMI distribution (E), smoking status and intensity (pack years) (F), metabolic comorbidities (G), and exercise activity (H).

(I, J) Dietary composition (I), and classification of diet into inflammatory and Western patterns (J).

(K, L) Ternary plots representing calculated macronutrient consumption of each patient per dietary classification.


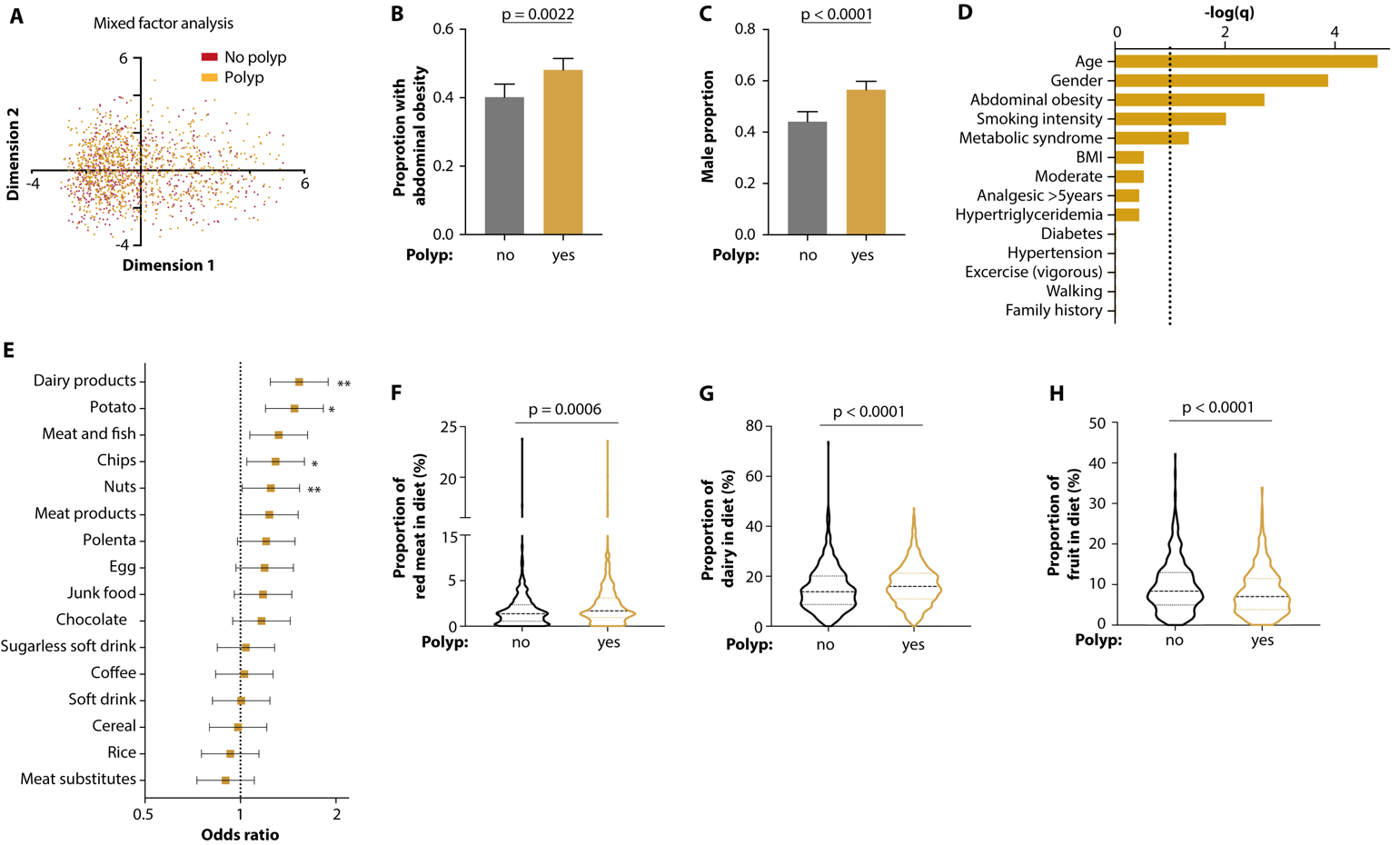


**Supplementary Results, Figure 2. Risk factor characterization of any polyp.**

(A) Mixed factor analysis-based dimensionality reduction plot of individuals based on their health, anthropometric, dietary, and lifestyle parameters.

(B, C) Comparison of various risk factors associated with polyp detection.

(D) Output from logistic regression models comparing individuals with any versus no polyps, adjusted for age, gender and BMI (dashed line represents significance threshold).

(E) Odds ratio plot with Wald 95% confidence interval for the indicated dietary components comparing any versus no polyp. Asterisks indicate results of the significance of logistic regression models of each risk factor, adjusted for age, gender and BMI.

(F-H) Comparison of various risk factors associated with polyp detection.

Two-tailed unpaired t-tests (B, C, F-H), Benjamini-Hochberg adjusted p-values from logistic regression models (E). * p<0.1, ** p<0.05, *** p<0.001, **** p<0.0001 was considered significant. Error bars (B, C) indicate mean with 95% confidence intervals.


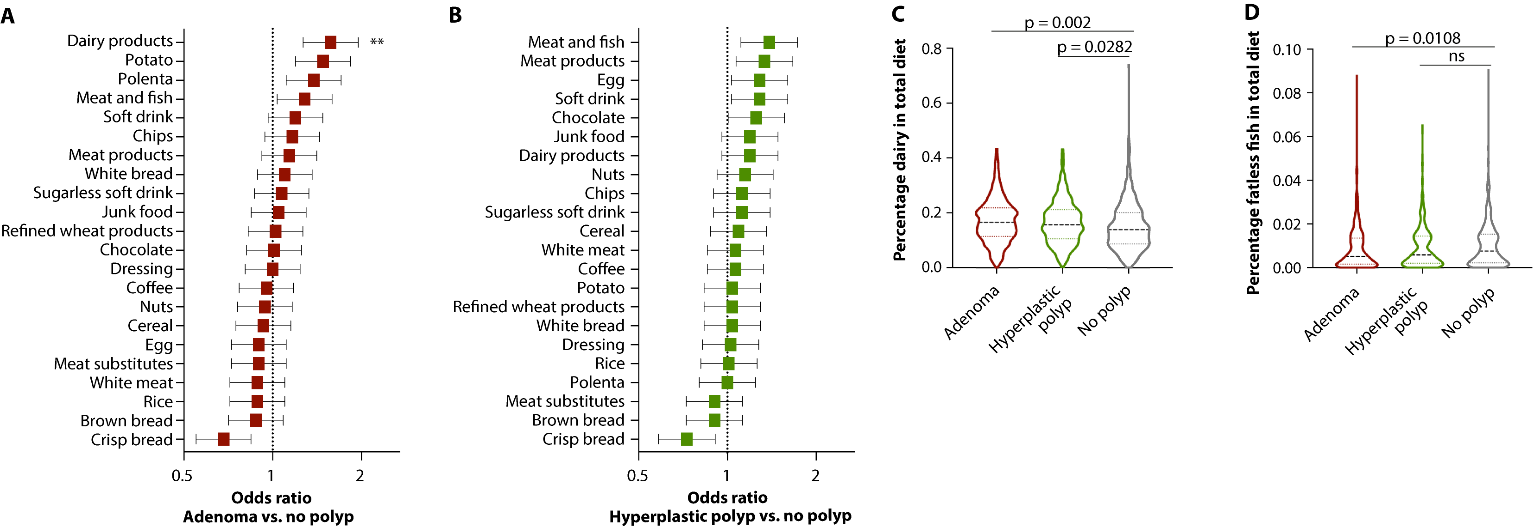


**Supplementary Results, Figure 3. Pathway-related risk factor characterization.**

(A, B) Odds ratio plots with Wald 95% confidence intervals for various dietary risk factors in the conventional (A) and alternative (B) pathway. Asterisks indicate results of the significance of logistic regression models of each risk factor, adjusted for age, gender and BMI.

(C, D) Comparison of various risk factors associated with the colonoscopy-based detection of adenomas, hyperplastic polyps, or neither.

Benjamini-Hochberg adjusted p-values from logistic regression models (A, B), ordinary one-way ANOVA with Turkey’s multiple comparisons (C, D). * p<0.1, ** p<0.05, *** p<0.001, **** p<0.0001 was considered significant.


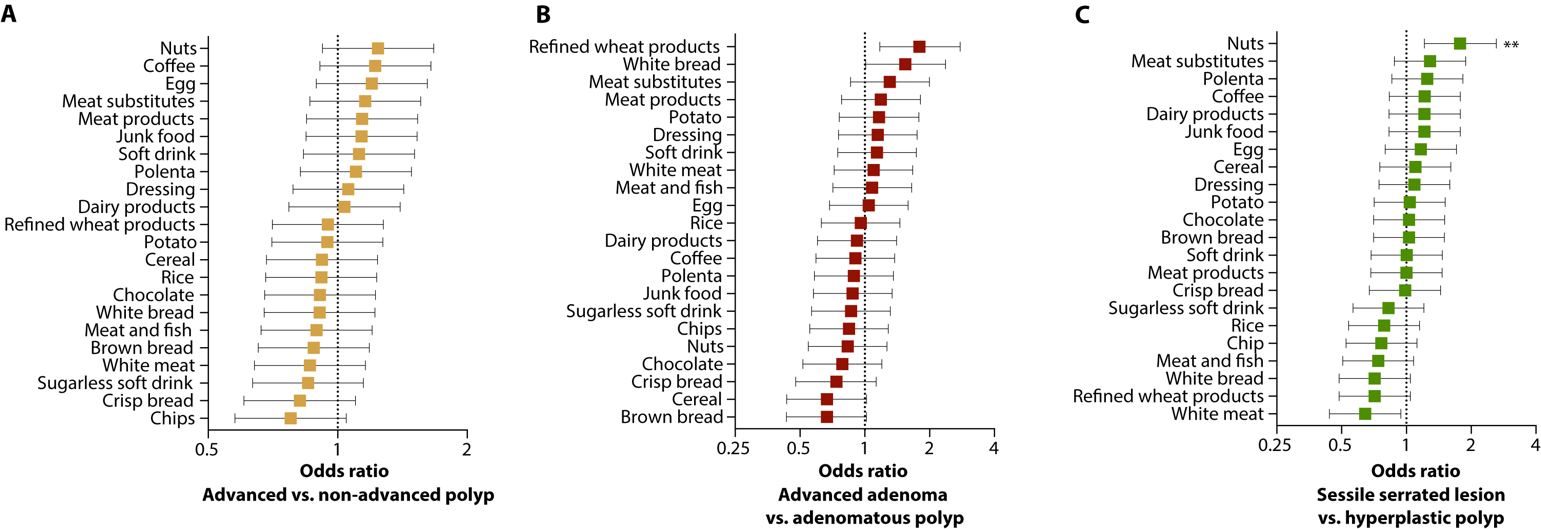


**Supplementary Results, Figure 4. Pathway-related risk factor characterization.**

(A-C) Odds ratio plots with Wald 95% confidence interval for various dietary risk factors for lesion progression overall (A), only in the conventional pathway (B), or only in the alternative pathway (C). Asterisks indicate results of the significance of logistic regression models of each risk factor, adjusted for age, gender and BMI.

Benjamini-Hochberg adjusted p-values from logistic regression (A-C). * p<0.1, ** p<0.05, *** p<0.001, **** p<0.0001 was considered significant.


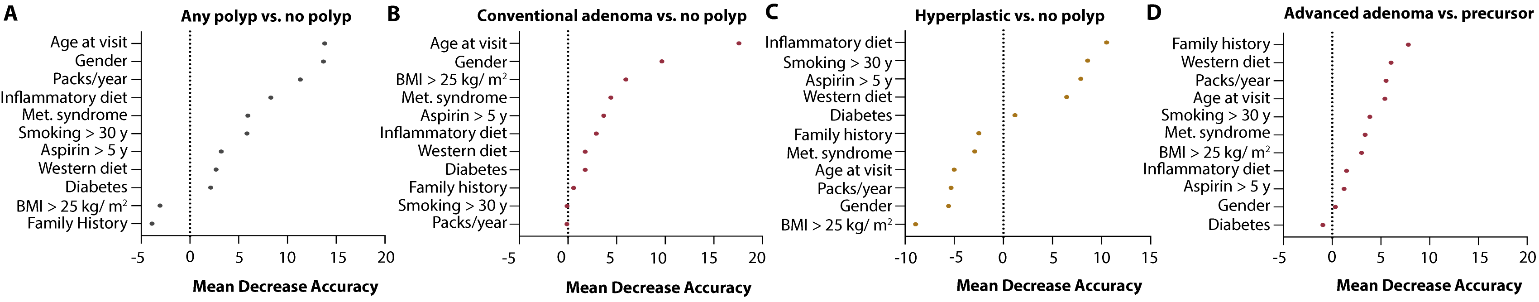


**Supplementary Results, Figure 5. Random forest classifier assessing risk factor contributions to lesion development and progression.**

(A-D) Mean accuracy decrease in a trained random forest models for each of the included model parameters when the risk factor is excluded. The model was applied to the prediction of any polyp (A), conventional adenomas (B), hyperplastic polyps (C), and advanced adenomas (D).

**Supplementary Results, Figure 6. Combinatorial effects of risk factors on polyp risk modulation.**

(A-F) Results of likelihood ratio tests, showing significance of the combined effects of any two risk factors in the classical (A-D) and serrated (E, F) pathways. Arrows indicate recommendations for risk mitigation (red arrows – risk exacerbation, green arrows – risk mitigation).

Benjamini-Hochberg adjusted p-values from likelihood ratio tests (A-F); dashed lines represent significance threshold. * p<0.1, ** p<0.05, *** p<0.001, **** p<0.0001 was considered significant.
